# Methanotrophic Poly(hydroxybutyrate) Through C1 Fermentation and Downstream Process Development: Molar Mass, Thermal and Mechanical Characterization

**DOI:** 10.3390/polym18020248

**Published:** 2026-01-16

**Authors:** Maximilian Lackner, Ľubomíra Jurečková, Daniela Chmelová, Miroslav Ondrejovič, Katarína Borská, Anna Vykydalová, Michaela Sedničková, Hamed Peidayesh, Ivan Chodák, Martin Danko

**Affiliations:** 1 CIRCE Biotechnologie GmbH, Kerpengasse 125, 1210 Wien, Austria; 2 Institute of Biology and Biotechnology, Faculty of Natural Sciences, University of Ss. Cyril and Methodius, 917 01 Trnava, Slovakia; jureckova1@ucm.sk (Ľ.J.); daniela.chmelova@ucm.sk (D.C.); miroslav.ondrejovic@ucm.sk (M.O.); 3 Polymer Institute, Slovak Academy of Sciences, Dúbravská cesta 9, 845 41 Bratislava, Slovakia; katarina.borska@savba.sk (K.B.); upolanvy@savba.sk (A.V.); michaela.sednickova@savba.sk (M.S.); hamed.peidayesh@savba.sk (H.P.); ivan.chodak@savba.sk (I.C.)

**Keywords:** gas fermentation, methanotroph, polyhydroxyalkanoates (PHA), solvent extraction, enzymatic treatment, process window

## Abstract

Today, PHB and its copolymers—potential plastic substitutes—are produced by fermenting sugar, which is not scalable to the volumes of plastic consumption. PHB from CH_4_ can offer a sustainable process route, with CH_4_ potentially produced from a variety of waste biomass streams through anaerobic digestion, gasification, and methanation. The high molar mass (M_w_) of PHB is a key determinant of its mechanical properties, and strain, culture conditions and downstream processing influence it. In this work, the strain *Methylocystis* sp. GB 25 (DSMZ 7674) was grown on natural gas as the sole carbon and energy source and air (1:1) in a loop reactor with 350 L active fermentation volume, at 35 °C and ambient pressure. After two days of continuous growth, the bacteria were limited in P and N for 1, 2, and 2.5 days to determine the optimal conditions for PHB accumulation and the highest Mw as the target. The biomass was then centrifuged and spray-dried. For downstream processing, chloroform solvent extraction and selected enzymatic treatment were deployed, yielding ~40% PHB from the biomass. The PHB obtained by solvent extraction exhibited high average weight molar masses of *M*_w_ ~1.1–1.5 × 10^6^ g mol^−1^. The highest *M*_w_ was obtained after one day of limitation, whereas enzyme treatment resulted in partially degraded PHB. Cold chloroform maceration, interesting due to energy savings, did not achieve sufficient extraction efficiency because it was unable to extract high-molar-mass PHB fractions. The extracted PHB has a high molar mass, more than double that of standard commercial PHB, and was characterized by DSC, which showed a high degree of crystallinity of up to 70% with a melting temperature of close to 180 °C. Mechanical tensile properties measurements, as well as dynamic mechanical thermal analysis (DMTA), were performed. Degradation of the PHB by enzymes was also determined. Methanotrophic PHB is a promising bioplastics material. The high M_w_ can limit and delay polymer degradation in practical processing steps, making the material more versatile and robust.

## 1. Introduction

Despite the many positive aspects of plastics, their direct and indirect impacts on the environment and human health have raised significant concerns, prompting the search for more sustainable alternatives. Among these, biobased and biodegradable polymers have been proposed, as they resemble conventional plastic materials in their processing and properties to a large extent. One class of such bioplastics is polyhydroxyalkanoates (PHA), which are naturally occurring polyesters. Short-, medium-, and long-chain length PHA have been discovered and developed. They can be formulated as copolymers and blends, providing a wide range of properties, which, together with the advantageous biodegradability of PHA in many ecosystems, including the marine environment, make them ideal candidates to replace fossil, non-degradable plastics. However, PHA is hardly available in the market today. There are only a handful of commercial manufacturers, with total production volumes less than 1/1000 of the standard plastics (while all other bioplastics together already have a 1–2% market share). One reason is that production is more costly than for commodity plastics such as polyolefins. Yet PHA is amongst the candidate materials with significant replacement potential for PE, PP, and other thermoplastics, and even elastomeric and thermoset materials. It can be assumed that PHA can replace up to 90% of conventional plastics (commodity and engineering materials) from a technical perspective, meaning that, in theory, 300 megatons/year would be required for that swap (leaving density differences aside). If the cost problems were solved, a second hurdle for PHA scale-up would remain: scalability. 1 kg of PHB, the most common PHA, requires approx. 2.8 kg of sugar for its manufacturing. So, if we were to replace 90% of 330 megatons/year of conventional plastics by PHB, we would need in excess of 900,000 km^2^ of arable land (at 10 tons of sugar/ha and year), which is more than 6.6% of all arable land [1] or the size of Nigeria or Venezuela. The global sugar production would have to be increased by a factor of more than 5.5 (from ~176 megatons/year) [2] to meet the raw material demand for that quantity of PHB. Such a scenario is not feasible. As 1st-generation biofuels have shown, competition over land resources at that scale will inevitably result in sharp increases in feed and food prices, which is not desired. Hence, alternative, more scalable feedstocks for large-volume PHA production are needed. One can think about lignocellulosic biomass, which is abundantly available. However, progress in enzymatic degradation to release sugar moieties has lagged behind expectations, despite long-lasting, significant research efforts. Methanotrophic bacteria can utilize CH_4_ as their sole carbon and energy source. A PHB yield of 0.230–0.487 g/g CH_4_ using mixed cultures derived from sewage sludge was demonstrated [3]. The global natural gas consumption is approximately 4500 billion m^3^/year. A quick, rough calculation (density of methane 0.66 kg/m^3^) shows that 21–44% of the global natural gas production volume would be needed to produce 300 megatons/year of PHB. When we assume that a circular plastics economy would recycle all plastics and that the material can withstand five mechanical recycling loops (thermal degradation during processing will degrade each plastic material from step to step), we can theoretically reduce the annual demand for PHA from 300 million tons to 60 million tons. That quantity would require 187–395 billion m^3^ of CH_4_, which is 5–10 times today’s biogas production volume (Based on [4]). The International Energy Agency (IEA)s World Energy Outlook 2023 sees the full potential of biogas and biomethane at 1000 billion m^3^ by 2050, with 300 billion m^3^ for biogas and biomethane production from agricultural residues and wastes near major pipelines, indicating that theoretically, enough PHA could be produced from that source to replace 90% of all conventional, fossil plastics. These considerations show that studying the production of PHA from methane is worthwhile. Microalgae offer another route to sustainable PHB; see, e.g., [5] about cyanobacteria for PHB production. Methanotrophs show higher growth rates than microalgae, making them more suitable for large-scale production.

PHB is produced and accumulated as an intracellular metabolite of producer cells [6]. The conventional approach to isolate PHB from cell biomass is chlorinated solvent extraction based on the solubilization of PHB in the cell and diffusion of this soluble fraction from the biomass [7,8,9]. The problem with this method of isolation is the need to dry the cell biomass, which incurs high energy costs, and the use of organic solvents, which pose both economic and environmental challenges for the technology [10]. An environmentally acceptable and economically more advantageous method appears to be the recovery of PHB by means of so-called non-PHA cell mass bio-digestion based on the enzymatic removal of the producer biomass components except the PHB biopolymer [11]. Different enzymes can be used for this purpose; however, as a common rule, researchers focus on enzymes that hydrolyze structural parts of the cell biomass such as proteins, carbohydrates and lipids [12,13,14,15].

In this article, we investigated the production of PHA (more specifically, PHB) from the methanotroph *Methylocystis* sp. GB 25 bacterial strain using CH_4_ as the sole carbon and energy source in a two-stage fed-batch fermentation with nutrient limitation (feast-famine cultivation strategy). A loop fermenter is better suited for gas fermentation than a conventional stirred tank reactor [16] and was, therefore, chosen for this work. Based on the above-mentioned downstream process possibilities, for PHB polyester, we applied simple chloroform extraction as an effective method suitable for high-molar-mass polyester, compared with enzyme-assisted extraction, a green alternative. High molar mass is crucial for mechanical strength and processing stability, yet challenging to preserve during extraction. Obtained PHB samples were characterized on a molecular level by gel permeation chromatography (GPC) and other physico-chemical characteristics, which were obtained from thermal and dynamic-mechanical analyses. These general parameters of PHB are necessary as a basis for the evaluation of its further processing conditions and applications.

## 2. Experimental Part

### 2.1. Materials and Methods

#### 2.1.1. Biomass Growth

For biomass growth, we used natural gas (>95% CH_4_) and air in a loop fermenter with a total volume of 400 L and an active fermentation volume of 350 L, with a pure culture of the strain *Methylocystis* sp. GB 25 (DSMZ 7674). The diameter of the fermenter was 20 cm in the riser and horizontal sections, and 30 cm in the downcomer; the flow speed was 2.5 m/s, and the ratio of natural gas/air was set at 1/1. The fermenter was held at 35 °C (±1 °C) and operated at ambient pressure. The gases were introduced via spargers, and the gas content of the fermentation broth (minimal medium) was 15%. For propulsion, a rim-driven thruster, as described earlier [17], was used. The fermenter was inoculated with 30 L. A stable cell density was achieved after 24 h. Traces of residual CH_4_ were disposed of by flaring. A description of the bioreactor is provided elsewhere [16]. We applied a two-stage “feast famine” regime, first growing the biomass with sufficient P and N for 2 days. Then, P and N were limited to 1, 2, and 2.5 days in the same fermenter and medium. The supply of CH_4_ and O_2_ has not changed. Samples were collected at the end of the three respective “famine” stages and centrifuged (2000 *g*, 5 min). These samples were named as PHB-1D, PHB-2D, and PHB-2.5D. The biomass was subsequently vacuum-dried (residual water content < 1%) and stored for downstream processing.

#### 2.1.2. Biomass Chloroform Extraction

The biopolymer PHB was extracted from the biomass using chloroform (p.a. grade, Microchem, Pezinok, Slovakia) in three consecutive extraction steps as follows: 5 g of biomass was extracted in a total of 500 mL of solvent (using 250 mL, 150 mL, and 100 mL in the first, second, and third extraction steps, respectively). The extraction was performed in a 500 mL round-bottomed flask equipped with a reflux condenser at chloroform reflux temperature and ambient pressure for 2 h. Residual biomass was separated by filtration using a sintered glass filter (the filter was washed with 10 mL of hot CHCl_3_), and the filtrate was used for the next extraction step. In the case of cold extraction (maceration) as an alternative extraction route, the experiment was carried out at laboratory temperature with constant stirring. The collected CHCl_3_ solution was concentrated to ~1/5 of its initial volume using a rotary vacuum evaporator. PHB was recovered by precipitation into 500 mL of methanol (p. a. grade, Microchem, Pezinok, Slovakia), filtered, and dried to constant weight. The PHB yield isolated from the dried biomass was calculated using Equation (1):

(1) $$\mathrm{PHB}\;(\mathrm{mg}/g)\;=\;\frac{m_{PHB}\;(\mathrm{mg})}{m_{biomass}\;(g)}$$

where *m_PHB_* is the weight of dried purified PHB and *m_biomass_* is the weight of the starting biomass.

#### 2.1.3. Enzymatic Treatment of Biomass

For enzymatic treatment, lyophilized *Methylocystis* sp. GB 25 biomass (sample PHB-1D) was mixed with 66 mM phosphate buffer, pH 7.4, at a ratio of 1:80 (*w*/*v*). After mixing, we sterilize the mixture at 121 °C for 20 min. After cooling to room temperature (r.t.), a 0.025% (*w*/*v*) solution of the respective enzyme in phosphate buffer, at a 1:2 (*v*/*v*) ratio to the biomass mixture volume, was added. The characteristics of the used enzymes were as follows: Trypsin—from porcine pancreas—2500 U/mg (VWR Chemicals, Solon, OH, USA); Lysozyme—from egg white—23,000 U/mg (VWR Chemicals, Solon, OH, USA); Lipase—from hog pancreas—23.9 U/mg (Sigma-Aldrich, Buchs, Switzerland). The formed mixture was added to the phosphate buffer to a final biomass content of 0.5% (*w*/*v*). The prepared reaction mixture was stirred and incubated for 24 h at 30 °C. After the enzymatic treatment period, the solid residue was separated from the mixture by centrifugation at 4000 rpm for 20 min. The supernatant was removed, and the sediment was washed with deionized water. After washing, the sediment was dried in an oven at 50 °C to constant weight. The biopolymer obtained dry weight after enzymatic treatment was evaluated gravimetrically. The PHB content in the dry biomass was determined by GC/FID (gas chromatography/flame ionization detector) analysis. The PHB purity in the dry biomass was calculated using Equation (2).

(2) $$\mathrm{PHB}\;\mathrm{in}\;\mathrm{biomass}\;(\%)=\frac{m_{PHB}\;(\mathrm{mg})}{m_{biomass}\;(g)}\;\times \;100$$

where *m_PHB_* is the weight of PHB determined by GC/FID, and *m_biomass_* is the weight of the dry biomass used for the determination of PHB by GC/FID.

#### 2.1.4. PHB Molecular Characterization

The GPC (gel permeation chromatography) of PHB polyesters was performed using an Agilent Technology 1260 Infinity pump equipped with a degasser, a thermostatic box for columns, and a refractive index detector (Azura 2.1 L RID, Knauer, Berlin, Germany). The partitioning system consisted of a PFG 7 μm pre-column and a linear PFG 7 μm particle-size column (d = 8 mm, l = 300 mm) (Agilent, Waldbronn, Germany), both thermostated at 40 °C. Elution was performed in a mixture of 2,2,2-trifluoroethanol (TFE) and 1,1,1,3,3,3-hexafluoro-2-propanol (HFIP) in a 9/1 (*v*/*v*) ratio, with the addition of 0.1 M potassium trifluoroacetate salt, at a flow rate of 1.0 mL min^−1^. Samples were filtered through a 0.22 µm pore size PTFE filter before injection. Polymethylmethacrylate narrow standards (Polymer Standard Services, Mainz, Germany) were used to construct the calibration. Commercial PHB (Biomer^®^, powder sample provided by Biomer (Krailling, Germany), with a density of 1.20 g/cm^3^) was used for comparison of molar masses as well as other thermal and physical parameters.

#### 2.1.5. Calorimetry

DSC (differential scanning calorimetry) analysis was performed on a DSC 821e Mettler Toledo (Mettler-Toledo, Greifensee, Switzerland) coupled with an intercooler. All measurements were performed under a nitrogen atmosphere with a flow rate of 50 mL/min. Each sample was heated from −20 to 200 °C at a rate of 5 °C/min, cooled to −20 °C, and then heated again. The melting temperatures were determined from the endothermic peak maxima of the 1st or 2nd heating runs using the STARe evaluation software from Mettler Toledo (Columbus, OH, USA). The enthalpic and temperature scales were calibrated using indium and zinc standards. The degree of crystallinity X_c_ (%) was calculated according to Equation (3):

X_c_ (%) = (∆H_m_/∆H_m_^R^) × 100 (3)

where ∆H_m_ is the melting enthalpy of the recovered PHB (J g^−1^), and ∆H_m_^R^ is the theoretical melting enthalpy of the 100% crystalline PHB, which is assumed to be 146.6 J g^−1^.

#### 2.1.6. Dynamic-Mechanical Thermal Analysis

Samples for DMTA with dimensions 10 × 7 × 1 mm^3^ were prepared by compression molding at 180 °C for 1 min. They were measured using the equipment DMA Q800 (TA Instruments, Hüllhorst, Germany) in tensile mode at a heating rate of 2 °C/min, a frequency of 10 Hz, and an amplitude of 20 µm. Samples were stored for 24 h and 14 days after compression molding before DMTA measurements. For each sample, two specimens were measured, and after various storage times, separate specimens were prepared for subsequent measurements.

#### 2.1.7. Tensile Test Analysis

The tensile properties were measured in dog-bone shaped test pieces with a 5 mm measuring section width and a 1 mm thickness. Testing was performed using an Instron 3365 (Instron, Norwood, MA, USA) universal testing machine in uniaxial deformation at a crosshead speed of 1 mm/min up to 1% deformation (to determine Young’s modulus), followed by a speed of 50 mm/min at higher deformations.

#### 2.1.8. Analysis of PHB by Gas Chromatography

PHB was evaluated in biomass and in extracted preparations by gas chromatography of methylated monomers after direct derivatization of the dry formulations.

From each sample, 5 mg was weighed into a vial, to which 2 mL of chloroform was added. Subsequently, we added 2 mL of acidified methanol, i.e., 3% (*v*/*v*) solution of H_2_SO_4_ in methanol. The reaction mixture was stirred and incubated at 100 °C for 2 h. After cooling to r.t., 1 mL of deionized water was added to the mixture. After mixing, we separated the two phases, and the lower phase (chloroform) was used for GC/FID analysis.

A sample with a volume of 2 µL was injected into an injector heated to 250 °C. The separation was carried out on a DB-23 capillary column (60 m × 0.25 mm) using nitrogen (N_2_) as carrier gas at a flow rate of 30 mL/min. The initial oven temperature, T_1_ = 80 °C, was maintained for 1 min. Subsequently, the temperature was increased at a rate of 8 °C/min to T_2_ = 200 °C, which was maintained for 5 min. The separated compounds were detected by an FID (flame ionization detector) at 270 °C. The concentration of PHB in the sample was calculated from the peak area at the retention time determined by evaluating standard solutions of PHB in the range of 1–10 g/L.

#### 2.1.9. Sampling and Statistical Evaluation of the Results

Samples obtained at different times of 1, 2, and 2.5 days of P and N limitation and marked as PHB-1D, PHB-2D, and PHB-2.5D were studied. Samples treated by trypsin and lysozyme enzymes or a mixture of enzymes with lipase were marked as PHB-1D/T, PHB-1D/Ly, PHB-1D/T + Ly, PHB-1D/T + Ly + L for sample PHB-1D treated by trypsin, lysozyme, trypsin + lysozyme, and trypsin + lysozyme + lipase, respectively. Each experiment was performed in three parallel measurements, with two repetitions. Sample PHBexp for tensile testing was obtained by mixing white powders of samples PHB-1D, PHB-2D, and PHB-2.5D each in 5 g to obtain 15 g of material for dog-bone specimens. Parameters of mechanical properties were obtained as averages with specific statistical errors from 7 tensile test measurements.

## 3. Results and Discussion

### 3.1. PHB Production

In contrast to classic fermentation with sugars, C1 fermentation uses gaseous feedstocks (CH_4_, CO, CO_2_) as the sole carbon source, with the main advantage being the fact that these can be derived from waste biomass, taking pressure from the agricultural system when large-scale production is envisioned. Selected methanotrophic bacteria, such as *Methylocystis* sp. GB 25 can be used to obtain PHB by first growing them with optimized supply of carbon (CH_4_), phosphorus (PO_4_^3−^) and nitrogen (NO_3_^−^ and/or HN_4_^+^) and then limiting them in P and N in the fermentation broth, through which the bacteria continue to assimilate carbon, but not as glycogen but in the form of PHB, as carbon storage compound. This strategy was employed in the current experiment, with a variation in the duration of this limitation period.

### 3.2. PHB Extraction

The biopolymer PHB was extracted from dried biomass using chloroform at a higher temperature. This PHB downstream process is considered the standard (laboratory) protocol [18]. Chloroform, before dichloromethane, is the most effective solvent for the highest extraction yield. At the same time, it has a relatively low impact on the undesired degradation or other physico-chemical properties of the PHB polyesters. The energetic, economic, and mainly environmental aspects, with the possibility to use greener solvents such as dimethyl carbonate [19,20,21] or 1,4-dioxolane [22], were not taken into consideration in this work, nor supercritical extraction with CO_2_ and cosolvents as previously described [23]. Three consecutive extraction steps at elevated temperature (chloroform reflux) were applied to extract bioplastics from biomass samples effectively. This ultimate extraction methodology was chosen to fully recover PHB, a prerequisite for a commercial process. Additionally, the pure residual biomass could be exploited for further purposes, e.g., as animal feed due to the high protein content. As is shown in Table S1 (Supporting Information), the obtained total yield of PHB from raw biomass was approx. 410–420 mg/g, and approx. 300, 100, and 15 mg/g were obtained on average in the first, second, and third extraction steps, respectively. Such a three-step elaborative procedure was necessary due to the high viscosity of the solutions (mainly in the first extraction step) under the used concentration conditions, suggesting a relatively high molar mass of PHB polyester. Following precipitation into methanol and drying allowed to obtain bright white PHB samples with a high purity of 98–99%. The content of PHB in biomass produced by methanotrophs could be even higher than in our case [24]. It was shown that technological production in a pressure reactor using methane as a carbon source with phosphorus, nitrogen, and ammonium deficiency could reach ~46–51% of PHB in biomass [25,26]. On the other hand, in the same technology, production with limitation on inorganic elements such as iron, potassium, magnesium, or sulfur gave a yield of PHB from biomass of 10 to 34% [25,27].

On the other hand, when cold chloroform extraction (maceration) was applied as milder alternative to prevent polyester degradation or to achieve energy savings, only totally approx. 300 mg/g in three consecutive extraction steps was obtained for sample PHB-2.5D. Compared to hot extraction, the polyester obviously remains partially in the biomass.

### 3.3. Enzymatic Treatment of Biomass

**Figure 1:**
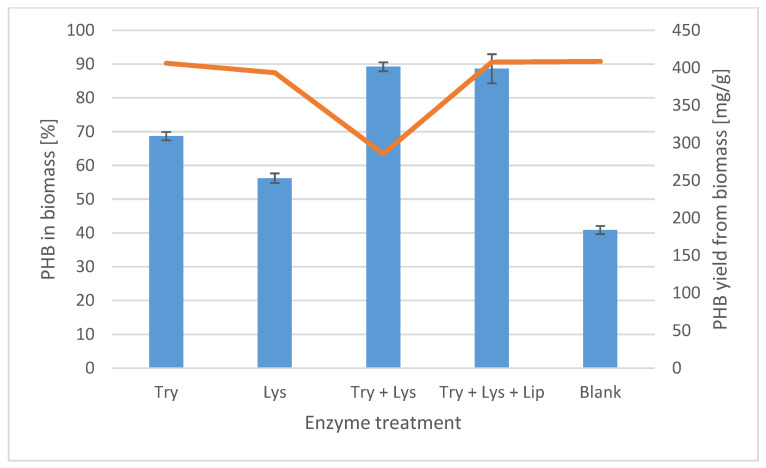
PHB is present in the biomass of *Methylocystis* sp. GB 25 after enzymatic treatment (blue bars) and PHB yield from biomass; Try—trypsin; Lys—lysozyme; Try + Lys—trypsin and lysozyme; Try + Lys + Lip—trypsin, lysozyme and lipase; Blank—original biomass without enzymatic treatment.

When processing producer biomass to isolate PHB from the intracellular space, specific hydrolytic enzymes targeting the individual polymeric structures comprising the cellular biomass can be used, focusing primarily on the structural parts of the biomass. Upon disruption of these parts, the cell’s intracellular contents are released into the surrounding solution. Proteinases (trypsin), glycosidases (lysozyme), and lipases are the most commonly used enzymes for cell lysis. While proteinases and glycosidases target essential cell wall components forming the vital barrier between the intercellular and extracellular space of the cell, and hence there is a significant reduction in the non-PHA portion of the biomass following disruption of these structures, lipases are more likely to have a supportive effect on the removal of cell membrane fatty acids and lipopolysaccharides forming the outer part of the cell wall of Gram-negative cells. Since *Methylocystis* sp. GB 25 is classified as a Gram-negative bacterium; we verified in our experiments the supporting role of lipases in the enzymatic treatment of bacterial biomass. The action of selected enzymes on bacterial biomass refinement for PHB isolation was studied using the commercially available enzymes trypsin, lysozyme, and lipase, as well as their combinations. Figure 1 shows the results of our enzymatic treatment experiment with sample PHB-1D.

Enzymatic treatment of the initial bacterial biomass resulted in the expected reduction in the non-PHA portions of the biomass, thereby increasing the PHB purity of the processed samples. However, the PHB purity in trypsin- and lysozyme-treated samples was only 69% and 57% (*w*/*w*), respectively. The best results were obtained with the combination of trypsin and lysozyme, and with the combination of trypsin, lysozyme, and lipase, in which the residue obtained by centrifugation of the mixture after enzymatic treatment contained 89 and 88% (*w*/*w*) PHB, respectively. It must be noted that this was achieved without the use of any chlorinated solvents. However, the two enzyme mixtures mentioned above yielded different biomass dry weights after centrifugation. In the case of the combination of both trypsin and lysozyme, we obtained approximately 286 mg/g of dry weight compared to the other treatments, where we obtained approx. 400 mg/g. The PHB yield of ~400 mg/g is close to the PHB yields of 410–420 mg/g obtained using solvent extraction. In this case, turbidity in the supernatant was observed after enzyme treatment. This suggests that the trypsin and lysozyme treatment increased the digestion of the non-PHA portion of the biomass; however, the released PHB was probably associated with lipophilic cell wall fragments and could not be fully accumulated in the pellet by centrifugation at 4000 rpm. The application of lipases confirmed the digestion efficiency of trypsin and lysozyme, and additionally, fatty acids were removed from the formed cell wall fragments, and PHB could be separated from the solution by centrifugation at 4000 rpm.

### 3.4. Molecular Characterization by GPC

**Figure 2:**
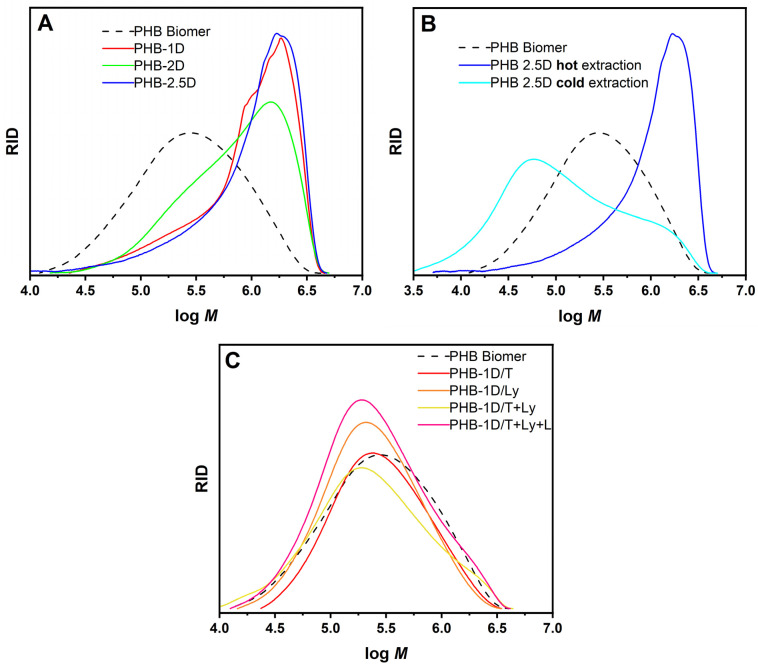
Molar mass distribution of PHB samples obtained from GPC analysis performed in TFE/HFIP/potassium trifluoro-acetate (9/1/0.1, *v*/*v*/wt%) elution solvent mixture: (**A**) comparison of samples obtained by hot chloroform extraction, (**B**) comparison with cold chloroform extraction, (**C**) obtained by different enzyme treatment.

The obtained PHB samples were characterized on the molecular level by gel permeation chromatography. The analysis was carried out in a solvent mixture of the fluorinated solvents TFE and HFIP to ensure the solubility of high-molar-mass PHB. The determined results of *M*_n_, *M*_w_ (number average molar mass, weight average molar mass), and Ɖ (dispersity index) obtained from GPC using calibration on PMMA standards are listed in Table 1 and Table 2. All studied PHB samples obtained by solvent extraction only had higher average molar masses than the commercial PHB (Biomer™) in the range of 10^6^ Da (Figure 2A). Such molar mass values are not exceptionally high for PHB produced by methanotrophs. *M*_w_ values ranging from 1.5 MDa [28] to 3.1 MDa [27] have been reported, depending on the preparation technology and nutrient limitation. *M*_w_ values of around 1.5 MDa have been obtained for a simple shaking reactor in the presence of citric acid and methanol [28], while *M*_w_ ~2 to 3 MDa have been obtained for PHB produced in pressure reactors with limitations of potassium, sulfur, iron, magnesium, nitrogen, phosphorus [27]. In our case, the ambient pressure technology used allowed obtained results comparable to those of the atmospheric reactor technology in both the compared parameters, yield and molar mass of PHB. Sample PHB-2D exhibited a somewhat broader molar mass distribution, with a long tail towards lower molar masses and with a hint of bimodal distribution, compared with the other samples, PHB-1D and PHB-2.5D. However, the fraction of lower molar masses in the range of 10^5^ Da was more or less pronounced in all samples. There is no clear trend in the comparison of samples toward degradation, broadening of the distribution, or the formation of a more significant fraction with high molar mass among PHB-1D, 2D, and 2.5D samples. A rather significant difference in molar mass of the obtained PHB is observed only when the cold extraction process (maceration) is applied (Figure 2B). The average value of *M*_w_ ~320 kDa for PHB was obtained for this process with broader dispersity Ɖ = 4.2. In such a case, the effectiveness of extracting high-molecular-mass ~10^6^ Da chains were lower, and more extraction steps were required.

On the other hand, all enzyme-treated samples exhibited lower molar masses (listed in Table 2) than the samples obtained by simple solvent extraction. These values were rather comparable to those of PHB from the commercial sample, with the molar mass distribution maxima slightly shifted to lower values, as shown in Figure 2C. This suggests that enzymatic treatment influences such high molar mass PHB biopolymer and, under the used conditions, causes its partial chain scission (degradation).

### 3.5. Thermal Properties Obtained from DSC

**Figure 3:**
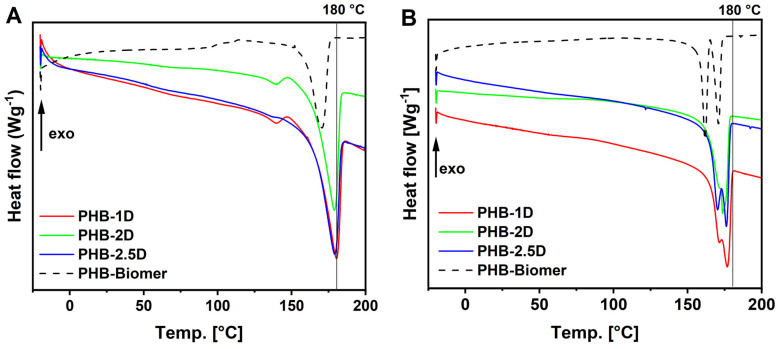

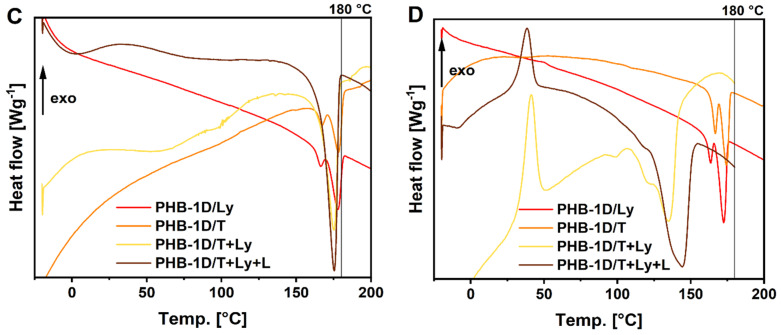
DSC 1st (**A**,**C**) and 2nd (**B**,**D**) heating runs of PHB samples obtained by solvent extraction (**A**,**B**) and enzyme treatment (**C**,**D**) downstream processes.

DSC analysis was performed to obtain thermal characteristics of PHB. The degree of crystallinity (X_c_) was calculated from the integrated melting endotherm of PHB crystals, using the heat of melting ∆H_m_. The melting temperatures (T_m_) were taken from the first heating runs and are listed in Table 1. Such PHB samples, equilibrated after precipitation and several hours of drying, can show higher X_c_ and T_m_ due to known physical aging of PHB. Values are more realistic and comparable to those obtained from the second heating runs, which depend on the cooling and heating rates. However, thermal characteristics from the second heating runs are shown in Figure 3B. The crystallinity degree values obtained from the single melting peak (Figure 3A) were similar across all samples and were close to Xc ~70%. Slightly lower values of 64–66% were then obtained from melting enthalpies of the second heating runs. These relatively high values exceeded the commercial PHB-Biomer™ value of ~60%. They were also much higher than the reported X_c_ ~36–57% for PHB recovered by DMC or CHCl_3_ from cyanobacteria-based biomass [21]. A similar trend was observed for T_m_, which ranged from 179 to 180 °C in our samples, whereas commercial PHB-Biomer™ exhibited a peak T_m_ of ~170 °C. The second heating run after cooling then showed a more complex melting endotherm, with two distinct melting peaks or shoulders, suggesting the formation of two types of PHB crystals (Figure 3B). In particular, the T_m_ were slightly shifted towards lower temperatures in the range of 170–177 °C (Table S2).

Enzyme-treated samples showed lower crystallite melting temperatures for both the 1st and 2nd heating runs. Samples obtained with a combination of enzymes also had significantly lower crystallinity degree X_c_, as shown by the values for the 1st and 2nd heating runs in Table 2 and Table S2. The situation is due to the fact that enzymatic treatment did not completely remove cell residues, and the PHB product was not purified by precipitation. The content of pure PHB in the enzyme-treated sample was up to 90%, as revealed in Figure 1. Biomass residues thus affect the crystallization of PHB and its thermal properties.

### 3.6. DMTA Characterization of the PHB

Dynamic-mechanical thermal analysis (DMTA) was used to determine the glass transition temperature T_g_, being the peak on the dependence of tan δ as storage (elastic) G′ and loss G″ moduli ratio (tan δ = G″/G’) on the temperature. The peaks in the tan δ curve correspond to transition temperatures associated with changes in the polymer’s physical structure, usually a type of movement typical of the particular structure. The most crucial parameter is the glass transition temperature T_g_, which exhibits the largest response with corresponding maxima. While other methodologies, including differential scanning calorimetry (DSC) and thermo-mechanical analysis (TMA), can be used to assess the glass transition, the DMTA technique is particularly sensitive as it directly measures molecular changes within the material. On the other hand, the calorimetrically measured thermal response in the glass transition for PHB is usually weak.

**Figure 4:**
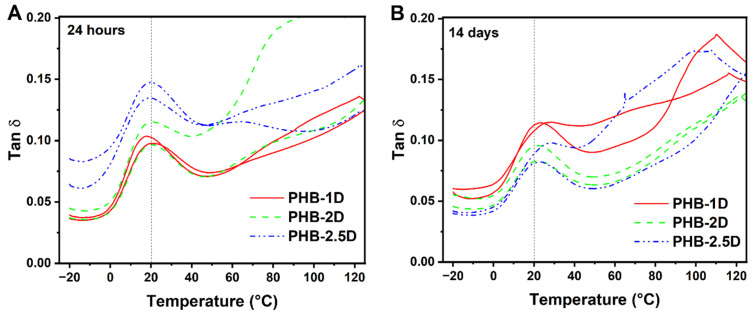
The tan δ dependence on temperature for PHB-1D (red), PHB-2D (green), and PHB-2.5D (blue) is shown for two independent measurements for (**A**) 24 h and (**B**) 14 days of sample storage.

The tan δ curves are shown in Figure 4, and the determined T_g_ values are listed in Table 3. The main peak in DMTA spectra appeared at approx. 20 °C and corresponds to T_g_.

However, differences in T_g_ are clearly observed between specimens measured after 24 h and those measured after 14 days of storage, with the latter showing a T_g_ 1–5 °C higher. Considering the characterization of PHB, an important factor is excessive physical aging. This phenomenon is observed in many plastics, but it is especially pronounced in polyhydroxyalkanoates. The effect consists of an extensive increase in Young’s modulus and an increase in brittleness of the polymer during storage, even at room temperature. The growth takes a few weeks, or even months, until a certain leveling off is seen. The first explanation is secondary crystallization, but parallel DSC measurements indicated only a marginal increase in the crystalline portion of 1–2%. A currently accepted explanation consists of the idea that the secondary crystallization occurs on long tie macromolecules in the amorphous phase, mainly in a large specific crystalline–amorphous interface. Secondary crystallization results in a higher stress on the tie macromolecules, which lose mobility and make the structure much more rigid and brittle, despite an almost negligible increase in total crystallinity [29].

Regarding the T_g_ values, the data show acceptable scatter, suggesting no change in the glass transition. This might also be expected, looking at the comparable molar masses of all three samples, we can conclude that the physical properties of the produced PHB do not depend on the fermentation conditions.

### 3.7. Tensile Test

Since we intended to assess the practical applicability of the newly prepared PHB, tensile mechanical properties were also tested and compared with those of the reference commercial PHB-Biomer. Additionally, we compared three different samples based on the fermentation process in the study, their similar molecular properties (*M*_w_) (Table 1) and T_g_ (Table 3), as well as storage moduli (data not given) obtained based on DMTA, indicate that there are no significant differences between them. For the evaluation of mechanical properties, we decided to mix all samples, i.e., PHB-1D, 2D, and 2.5D into one sample (named PHBexp) for preliminary mechanical properties assessment of the obtained PHB. The parameters, such as tensile strength, strain at break, and Young’s modulus, are evaluated for PHA polyesters as standard after a certain storage period to evaluate the impact of physical aging. Here, we determined these parameters by measuring the mechanical properties after 24 h and 14 days. The data is shown in Table 3.

The data indicate that all three parameters are higher for the PHB-Biomer than for the PHBexp sample, despite the PHBexp’s higher molar mass, crystallinity, and T_g_. Moreover, physical aging after two weeks of storage did not affect the tensile strength. Still, it resulted in a statistically significant decrease in strain at break, accompanied by an increase in Young’s modulus, while the parameters remained lower for PHBexp than for commercial PHB-Biomer. The same effect was reported in other published reports [30]. The reason may be substantially higher PHBexp molar mass (*M*_w_ > 10^6^ Da) compared to PHB-Biomer, which is approx. three times lower, as briefly discussed below.

Under sample preparation conditions (high-temperature treatment at 180 °C for 1 min), PHB may undergo partial degradation, see Table 4. These conditions are dictated by the high melting temperature of PHB. Exact determination of molar masses showed that the lower molar mass PHB-Biomer was relatively stable compared to PHBexp. From this point of view, a decrease in molar mass due to degradation at 180 °C affects PHB with higher molar masses more, which is understandable given the statistically higher probability of thermally triggered scission in longer chains. Under sample preparation conditions, the average decrease in the molar mass of PHB-Biomer was only 5%, whereas for PHBexp the decrease was 25%. The lower thermal stability of PHBexp and the corresponding higher degree of thermal degradation compared to reference PHB are due to an increase in the number of defects in the supramolecular structure of PHB, especially in the tie macromolecular segments connecting the amorphous and crystalline phases, which keep the whole structure together when load is applied. The defects, formed during thermal degradation, act as sites that facilitate deformation and, especially, as initiation sites for catastrophic crack formation, resulting in lower strain at break and making the material more brittle.

## 4. Conclusions

The present work demonstrates the successful optimization of PHB production by methane-utilizing *Methylocystis* sp. GB 25 through the fine-tuning of both fermentation and downstream processing parameters. Our findings confirm that not only the choice of microbial strain and fermentation conditions, but also the selection and optimization of downstream processing steps exert a decisive influence on the molecular properties of the resulting PHB, in particular its molar mass and crystallinity—key parameters for its mechanical performance and processability.

A major achievement of this study was the production of high-molar-mass PHB (*M*_w_ up to ~1.5 × 10^6^ g mol^−1^), significantly surpassing commercial PHB benchmarks and providing materials with enhanced crystallinity (~70%) and elevated melting temperatures (~180 °C). The three limitation times did not yield significantly different PHB. Thermal processing will always lead to partial degradation, but it is more pronounced for high molar mass PHB.

Enzymatic treatments proved less effective at maintaining high molar mass PHB. In addition, enzymatic treatment offers an environmentally favorable alternative to solvent extraction by avoiding chlorinated solvents; partial degradation of the polymer chains was observed, resulting in significantly reduced molar masses closer to those of commercial grades. This observation underscores the trade-offs between green extraction strategies and polymer quality when targeting high-performance applications.

Moreover, this study adds to the growing body of evidence that methanotrophic PHB can match or exceed the material quality of sugar-based PHB, reinforcing the potential of gas fermentation as a scalable, non-food-competing route for producing bioplastics.

This work provides empirical data and practical insights for advancing the transition from laboratory-scale studies to industrial production of PHB from methane. It highlights the feasibility of producing premium-grade biopolyesters through gas fermentation, though at this stage it relies on traditional solvent extraction methods to achieve the highest product quality. The observation of bimodal molar mass distribution is also highly interesting: low *M*_w_ polymer chains enhance processability, while high *M*_w_ ones determine the mechanical properties such as tensile strength. This combination is sometimes achieved through blending, which could be avoided by this intrinsic material property.

## 5. Outlook

While this study has demonstrated the technical feasibility of producing high-molar-mass PHB from methane with properties comparable to or exceeding those of conventional PHB, several aspects warrant further investigation to enable industrial scalability and broader adoption.

The downstream processing of PHB remains a critical bottleneck. Additionally, chloroform extraction is effective for laboratory-scale purification; its scalability is constrained by environmental and regulatory concerns. Future research must focus on developing greener, scalable extraction techniques, potentially employing emerging solvent systems (e.g., cyclic carbonates, ionic liquids, deep eutectic solvents) or hybrid processes combining physical, enzymatic, and chemical approaches. The use of non-halogenated, low-impact solvents or solvent-free methods aligned with circular economy principles will be crucial to meeting sustainability targets.

The application of process modeling and digital tools, such as machine learning or advanced process control, could offer significant benefits for optimizing fermentation conditions in real time. Tailoring nutrient limitation strategies dynamically based on in-line monitoring could further enhance PHB yield and tailor polymer properties to specific application needs.

From a materials perspective, the exploration of PHB blends or copolymers derived from methane fermentation (e.g., PHBV or PHBH) offers promising avenues to broaden the property spectrum beyond what pure PHB can provide. These materials could address known limitations of PHB, such as brittleness and narrow processing windows, further enhancing market acceptance.

The integration of methane-based PHB production into existing industrial infrastructures (e.g., biogas plants, wastewater treatment facilities) represents a strategic opportunity for decentralized, sustainable bioplastics manufacturing. This alignment with circular bioeconomy concepts would valorize waste streams, reduce greenhouse gas emissions, and decouple bioplastics production from agricultural feedstocks.

There is also an urgent need to develop fully biobased and biodegradable additives for bioplastic materials such as PHB, for nucleation, stabilization, and enhanced properties.

While the technical proof-of-concept has been established, the path to industrial application of methane-derived PHB demands continued interdisciplinary efforts across microbiology, process engineering, polymer science, and sustainability assessment. The potential environmental benefits and scalability make this a compelling avenue for future bioplastic production.

Except for the higher price of polyhydroxyalkanoates compared to high-volume thermoplastics, a main limitation and disadvantage of PHA consists of low thermal resistance, expressed by rather fast degradation of the materials due to breaking the bonds of the main chains of the polymer. Therefore, in practically all cases, it is recommended to process the material at the lowest possible temperature and in the shortest processing time. In the past, we made a thorough investigation of the temperature effect on the decrease in molecular weight and found that even at a temperature of 130 °C (well below the melting point of PHB), a 10% molecular weight decrease occurs within 10 min. The standard processing temperature must be at least 180 °C or higher, i.e., above the melting point, inevitably harming (degrading) the PHA. Thus, a high *M*_W_ of the PHAs after production is desirable to have a high enough molar mass after processing. While in this work, we did not deal with the particular (tarachieve) application of the PHB, the material usually has excellent ultimate physical properties in spite of certain degradation during the final step of processing. It could substitute a number of polymers made from fossil raw materials, depending on the physical properties of the available biodegradable PHA. The main problem occurs if blow molding processing is applied, since compression molding and injection molding can be adjusted to acceptable processing conditions regarding temperature and time; in the case of blow molding, the material after a rather small decrease in molar mass may have too small a strength of the melt so that the extruded sleeve will break when blowing to a lower foil thickness. This disadvantage may be partially counteracted by a molar weight well over one million so that even after substantial degradation, the material still can be processed. Another way of utilizing the high MW of our novel PHA material may consist of the preparation of oriented materials, e.g., fibers or foils. These products made from standard commercial PHA might be difficult to draw due to a fast decrease in molar mass with starting *M*_w_ below, often say 400,000 D.

## Figures and Tables

**Table 1:** Determined average molar masses M_n_ and M_w_ and dispersity index Ɖ from GPC and melting temperature and crystallinity degree obtained from 1st heating DSC analysis of samples obtained by chloroform extraction.

Sample	*M*_n_ ^a^ × 10^3^ (g mol^−1^)	*M*_w_ ^a^ × 10^3^ (g mol^−1^)	Ɖ ^a^	T_m1_^b^ °C	T_m2_^b^ °C	∆H_m_^b^ (Jg^−1^)	X_c_^c^ (%)
PHB-1D	758.6	1504.2	2.00	-	180.5	102.23	70.00
PHB-2D	409.1	1101.5	2.69	-	179.0	101.18	69.30
PHB-2.5D	413.6	1407.0	3.39	-	179.3	102.06	69.90
PHB-Biomer^®^	165.1	487.1	2.95	-	171.2	87.94	60.00

^a^ Determined from GPC using 1 mL/min flow of TFE/HFIP, 9/1 eluent using PMMA narrow standard; ^b^ obtained from DSC 1st heating run; ^c^ calculated according to Equation (3).

**Table 2:** Determined average molar masses M_n_ and M_w_ and dispersity index Ɖ from GPC and melting temperature and crystallinity degree obtained from 1st heating DSC analysis of sample PHB-1D treated by different enzyme mixture.

Sample PHB-1D/ Enzyme	*M*_n_ ^a^ × 10^3^ (g mol^−1^)	*M*_w_ ^a^ × 10^3^ (g mol^−1^)	Ɖ ^a^	T_m1_^b^ °C	T_m2_^b^ °C	∆H_m_^b^ (Jg^−1^)	X_c_^c^ (%)
PHB-1D/T	206.2	479.0	2.32	167.5	178.2	76.89	52.45
PHB-1D/Ly	166.9	406.8	2.43	166.2	177.6	62.60	42.70
PHB-1D/T + Ly	134.2	464.2	3.46	-	175.2	68.09	46.45
PHB-1D/T + Ly + L	160.7	464.0	2.88	-	175.4	73.00	49.80

^a^ determined from GPC using 1 mL/min flow of TFE/HFIP, 9/1 eluent using PMMA narrow standard; ^b^ obtained from DSC 1st heating run; ^c^ calculated according to Equation (3).

**Table 3:** Determined T_g_ based on DMTA and Mechanical properties (tensile strength at break σ_B_, strain at break ε_B_, and Young’s modulus E) of PHB, comparison of PHBexp with PHB-Biomer, after 24 h and 14 days of storage.

Sample	σ_B_, MPa		ε_B,_ %		E, MPa		T_g_^(DMTA)^, °C	
	24 h	14 d	24 h	14 d	24 h	14 d	24 h	14 d
PHB-1D	-	-	-	-	-	-	20.5	26.0
PHB-2D	-	-	-	-	-	-	20.9	22.1
PHB-2.5D	-	-	-	-	-	-	19.6	23.4
PHBexp	38.3 ± 2.3	38.0 ± 1.5	2.9 ± 0.3	2.0 ± 0.2	3523 ± 148	4370 ± 107	-	-
PHB-Biomer^®^	53.6 ± 2.4	55.4 ± 1.6	3.4 ± 0.3	2.8 ± 0.1	4122 ± 384	4870 ± 184	19.4	

**Table 4:** Molecular characteristics of PHBexp and PHB Biomer samples before and after thermal processing by compression molding at 180 °C for 1 min.

Sample	Before		After		Change
	*M*_w_ ^a^ × 10^3^ (g mol^−1^)	Ɖ ^a^	*M*_w_ ^a^ × 10^3^ (g mol^−1^)	Ɖ ^a^	%
PHBexp	1 135.5	1.98	854.0	1.91	−25
PHB Biomer	487.1	2.95	462.7	1.95	−5

^a^ Determined from GPC using 1 mL/min flow of TFE/HFIP, 9/1 eluent using PMMA narrow standard.

## Data Availability

The raw data supporting the conclusions of this article will be made available by the authors on request.

## Supplementary Materials

The following supporting information can be downloaded at: https://www.mdpi.com/article/10.3390/polym18020248/s1, Table S1: Experimental details of PHB chloroform extraction from dry biomass; Table S2: DSC based results obtained from 2nd heating run.

## References

[1] World Population Review. https://worldpopulationreview.com/country-rankings/arable-land-by-country.

[2] ISO (International Sugar Organization) (2024). https://www.isosugar.org/sugarsector/sugar.

[3] Zhang T.T., Wang X.W., Zhou J.T., Zhang Y. (2018). Enrichments of methanotrophic–heterotrophic cultures with high poly-β-hydroxybutyrate (PHB) accumulation capacities. J. Environ. Sci..

[4] Enerdata (2024). https://yearbook.enerdata.net/natural-gas/world-natural-gas-production-statistics.html.

[5] Lackner M., Kamravamanesh D., Krampl M., Itzinger R., Paulik C., Chodak I., Herwig C. (2019). Characterization of photosynthetically synthesized poly(3-hydroxybutyrate) using a randomly mutated strain of *Synechocystis* sp. PCC 6714. Int. J. Biobased Plast..

[6] Anderson A.J., Dawes E.A. (1990). Occurrence, metabolism, metabolic role, and industrial uses of bacterial polyhydroxyalkanoates. Microbiol. Rev..

[7] Aramvash A., Moazzeni-Zavareh F., Gholami-Banadkuki N. (2018). Comparison of different solvents for extraction of polyhydroxybutyrate from *Cupriavidus necator*. Eng. Life Sci..

[8] Jiang G., Johnston B., Townrow D., Radecka I., Koller M., Chaber P., Adamus G., Kowalczuk M. (2018). Biomass extraction using non-chlorinated solvents for biocompatibility improvement of polyhydroxyalkanoates. Polymers.

[9] Didion Y.P., Vargas M.V.G.A., Tjaslma T.G., Woodley J., Nikel P.I., Malankowska M., Su Z., Pinelo M. (2024). A novel strategy for extraction of intracellular poly(3-hydroxybutyrate) from engineered *Pseudomonas putida* using deep eutectic solvents: Comparison with traditional biobased organic solvents. Sep. Purif. Technol..

[10] Koller M. (2020). Established and advanced approaches for recovery of microbial polyhydroxyalkanoate (PHA) biopolyesters from surrounding microbial biomass. EuroBiotech J..

[11] Pagliano G., Galletti P., Samorì C., Zaghini A., Torri C. (2021). Recovery of polyhydroxyalkanoates from single and mixed microbial cultures: A review. Front. Bioeng. Biotechnol..

[12] de Koning G., Witholt B. (1997). A process for the recovery of poly(hydroxyalkanoates) from Pseudomonads Part 1: Solubilization. Bioprocess Eng..

[13] Kapritchkoff F.M., Viotti A.P., Alli R.C.P., Zuccolo M., Pradella J.G.C., Maiorano A.E., Miranda E.A., Bonomi A. (2006). Enzymatic recovery purification of polyhydroxybutyrate produced by *Ralstonia eutropha*. J. Biotechol..

[14] Neves A., Müller J. (2012). Use of enzymes in extraction of polyhydroxyalkanoates produced by *Cupriavidus necator*. Biotechnol. Progr..

[15] Gutt B., Kehl K., Ren Q., Boesel L.F. (2016). Using ANOVA models to compare and optimize extraction protocols of P3HBHV from *Cupriavidus necator*. Ind. Eng. Chem. Res..

[16] Safaeian P., Yazdian F., Khosravi-Darani K., Rashedi H., Lackner M. (2023). P3HB from CH4 using methanotrophs: Aspects of bioreactor, fermentation process and modelling for cost-effective biopolymer production. Front. Bioeng. Biotechnol..

[17] Lackner M., Löhr A., Schill F., Van Essche M. (2024). Rim Driven Thruster as Innovative Propulsion Element for Dual Phase Flows in Plug Flow Reactors. Fluids.

[18] Koller M., Niebelschutz H., Braunegg G. (2013). Strategies for recovery and purification of poly[(*R*)-3-hydroxyalkanoates] (PHA) biopolyesters from surrounding biomass. Eng. Life Sci..

[19] Mongili B., Azim A.A., Garofalo S.F., Batuecas E., Re A., Bocchini S., Fino D. (2021). Novel insights in dimethyl carbonate-based extraction of polyhydroxybutyrate (PHB). Biotechnol. Biofuels.

[20] Abbasi M., Coats E.R., McDonald A.G. (2022). Green solvent extraction and properties characterization of Poly(3-hydroxybutyrate-co-3-hydroxyvalerate) biosynthesized by mixed microbial consortia fed fermented dairy manure. Bioresour. Technol. Rep..

[21] Yashavanth P.R., Maiti S.K. (2024). Recovery and characterization of polyhydroxybutyrate from *Chlorogloea fritschii* TISTR 8527 using halogenated and green solvents. J. Appl. Phycol..

[22] Wongmoon C., Napathorn S.C. (2022). Optimization for the efficient recovery of poly(3-hydroxybutyrate) using the green solvent 1,3-dioxolane. Front. Bioeng. Biotechnol..

[23] Nayir T.Y., Konuk S., Kara S. (2023). Extraction of polyhydroxyalkanoate from activated sludge using supercritical carbon dioxide process and biopolymer characterization. J. Biotechnol..

[24] Liu L.-Y., Xie G.-J., Xing D.-F., Liu B.-F., Ding J., Ren N.-Q. (2020). Biological conversion of methane to polyhydroxyalkanoates: Current advances, challenges, and perspectives. Environ. Sci. Ecotechnol..

[25] Wendlandt K.-D., Jechorek M., Helm J., Stattmeister U. (2001). Producing poly-3-hydroxybutyrate with a high molecular mass from methane. J. Biotechnol..

[26] Wendlandt K.-D., Jechorek M., Helm J., Stattmeister U. (1998). Production of PHB with a high molecular mass from methane. Polym. Degrad. Stab..

[27] Helm J., Wendlandt K.-D., Jechorek M., Stattmeister U. (2008). Potassium deficiency results in accumulation of ultra-high molecular weight poly-β-hydroxybutyrate in a methane-utilizing mixed culture. J. Appl. Microbiol..

[28] Zhang Y., Xin J., Chen L., Song H., Xia C. (2008). Biosynthesis of poly-3-hydroxybutyrate with a high molecular weight by methanotroph from methane methanol. J. Nat. Gas Chem..

[29] de Koning G.J.M., Scheeren A.H.C., Lemstra P.J., Peeters M., Reynaers H. (1994). Crystallization phenomena in bacterial poly[(R)-3-hydroxybutyrate]: 3. toughening via texture Changes. Polymer.

[30] Chodák I., Scott G. (2002). Polyhydroxyalkanoates: Properties and Modification for High Volume Applications, Degradable Polymers, Principles and Applications.

