# Supplementary material for: Methanotrophic Poly(hydroxybutyrate) Through C1 Fermentation and Downstream Process Development: Molar Mass, Thermal and Mechanical Characterization
Source: Polymers (Basel). 2026 Jan 16;18(2):248. doi: 10.3390/polym18020248 (PMC12846098; doi:10.3390/polym18020248)
Supplement: Supplementary file 1 [file polymers-18-00248-s001.zip › polymers-4001179-supplementary.pdf]

Supporting Information

Methanotrophic Poly(hydroxybutyrate) through C1 Fermentation and Downstream Process Development: Molar Mass, Thermal and Mechanical Characterization

Maximilian Lackner[1,*], Ľubomíra Jurečková[2], Daniela Chmelová[2], Miroslav Ondrejovič[2], Katarína Borská[3], Anna Vykydalová[3], Michaela Sedničková[3], Hamed Peidayesh[3], Ivan Chodák[3], Martin Danko[3,*]

[1] CIRCE Biotechnologie GmbH, Kerpengasse 125, 1210 Wien, Austria

[2] Institute of Biology and Biotechnology, Faculty of Natural Sciences, University of Ss. Cyril and Methodius, 917 01 Trnava, Slovakia

[3] Polymer Institute, Slovak Academy of Sciences, Dúbravská cesta 9, 845 41 Bratislava, Slovakia

*Correspondence to: m.lackner@circe.at; martin.danko@savba.sk

Table S1: Experimental details of PHB chloroform extraction from dry biomass.

Sample	Biomass Starting weight (g)	Extraction step 1 in 250 ml	Extraction step 2 in 150 ml	Extraction step 3 in 100 ml	Total PHB weight, (g)
PHB-1D	5.02	1.47 (29.2%)	0.57 (11.4%)	0.068 (1.4%)	2.04 (41%)
PHB-2D	5.01	1.69 (33.7%)	0.35 (7.0%)	0.065 (1.3%)	2.11 (42%)
PHB-2.5D	5.02	1.51 (30.1%)	0.44 (8.8%)	0.1 (2.1%)	2.05 (41%)

Table S2: DSC based results obtained from 2nd heating run.

**Sample**	T_g °C	T_c °C	T_{m1} °C	T_{m2} °C	ΔH_m (Jg^{-1})	X_c[a] (%)
PHB-1D	4.8	-	171.4	176.8	93.78	64.20
PHB-2D	4.1	-	170.2	173.6	95.84	65.64
PHB-2.5D	6.8	-	170.2	176.2	93.50	64.04
PHB-1D/T	-	-	166.1	174.4	64.40	44.11
PHB-1D/Ly	-	-	163.7	172.4	60.03	41.12
PHB-1D/T+Ly	-	41.1	119.0	135.2	40.91	27.91
PHB-1D/T+Ly+L	-	38.5	-	144.1	54.33	37.06
PHB-Biomer®	5.5	-	161.7	171.0	91.30	62.28

[a] calculated according to Eq.: X_c (%) = $(\Delta H_m/\Delta H_m^R) \times 100$, $\Delta H_m^R = 146.6$ J g^{-1}.
